# Neurologic manifestations of COVID-19 infection in Asia: a systematic review

**DOI:** 10.1186/s41983-021-00279-3

**Published:** 2021-02-17

**Authors:** I. Putu Eka Widyadharma, Alvin Hendellyn, A. A. A. Putri Laksmi Dewi, I. Made Oka Adnyana, D. P. G. Purwa Samatra, Desak Ketut Indrasari Utami

**Affiliations:** Department of Neurology, Faculty of Medicine, Udayana University/Sanglah General Hospital, Bali, Indonesia

**Keywords:** Neurologic manifestation, COVID-19, Systematic review

## Abstract

**Background:**

COVID-19 infection can show various manifestation, including neurologic manifestations, such as *anosmia*, *ageusia*, or *dysgeusia*, and causes the neurologic disorder such as stroke, Guillain-Barre syndrome, encephalopathy, and many more.

**Aim:**

To briefly review neurologic manifestation in COVID-19 infection in the Asia region (South East Asia and the Western Pacific Region).

**Material and methods:**

This review uses the PRISMA statement and checklist. The source for reviewed article was performed in PubMed that were published between December 2019 to September 2020 with the latest 1 year of publication. Study titles were first screened, then reviewed by title and abstract and then the last review, we tested full text and applied eligibility criteria.

**Results:**

We found a total of 9 retrieved articles from the electronic database. Among these 9 articles, 5 of them are case report, 1 case series, 1 prospective multi-center cohort study, 1 retrospective multi-center study, and 1 retrospective observational study. All articles reported confirmed COVID-19, confirmation by positive swab test using the real-time RT-PCR method, with neurologic manifestations, disorder, or syndrome on presentation or found during hospital stay. In case of neurologic disorder or syndrome, the studies reported encephalitis and ADEM, acute cerebrovascular disease, acute symptomatic seizure, and Guillain-Barré syndrome with acute cerebrovascular disease as the most common neurologic disorder associated with COVID-19 infection, followed by encephalitis.

**Conclusion:**

COVID-19 also affects the brain, which may result in a global or focal neurologic manifestation. Healthcare provider treating patient with COVID-19 infection should also be aware of neurologic manifestation associated with COVID-19 infection to improve patient’s outcome.

Guillain-Barre syndrome, encephalopathy, and many more. This review will briefly review neurologic manifestation in COVID-19 infection in the Asian region (South East Asia and the Western Pacific Region. A total of 9 retrieved articles from the electronic database reported confirmed COVID-19, confirmation by RT-PCR method, with neurologic manifestation, disorder, or syndrome on presentation or found during hospital stay. Healthcare provider treating patient with COVID-19 infection should also be aware of neurologic manifestation associated with COVID-19 infection to improve patient’s outcome.

## Introduction

COVID-19 or coronavirus disease 2019 is viral infection caused by coronavirus. Based on its phylogenetic test, it is included in the same subgenus as coronavirus, which caused the SARS outbreak in 2002–2004, Sarbecoronavirus. This disease started from pneumonia of unknown etiology in Wuhan, China, in December 2019 and has spread worldwide since then and now stated as a pandemic in most countries in the world, including Asian countries [1].

WHO global situation reports on 27 September 2020 reported 33 million confirmed cases with 996,342 deaths in the world [2]. South-East Asia is the second-highest region of a confirmed case, after Americas, with 6,720,771 (21%) cases and the third-highest of death with 111,711 (11%) death [2]. Western Pacific region consists of other Asian countries, the region with the lowest confirmed case and death with 600,891 (2%) and 13.129 (1%) death [2].

COVID-19 infection can show various manifestations, from the usual viral infection symptoms, including dry cough, malaise, and fever to a more severe symptoms like shortness of breath. It has been reported that COVID-19 infection can also show various neurologic manifestations, such as *anosmia*, *ageusia*, or *dysgeusia*, and causes the neurologic disorder such as stroke, Guillain-Barre syndrome, encephalopathy, and many more. This indicates that clinicians have to be more aware when dealing with COVID-19 suspected patients presenting with neurologic deficits.

This article will briefly review neurologic manifestation in COVID-19 infection in the Asian region (South East Asia and the Western Pacific Region).

## Materials and methods

**Fig. 1:**
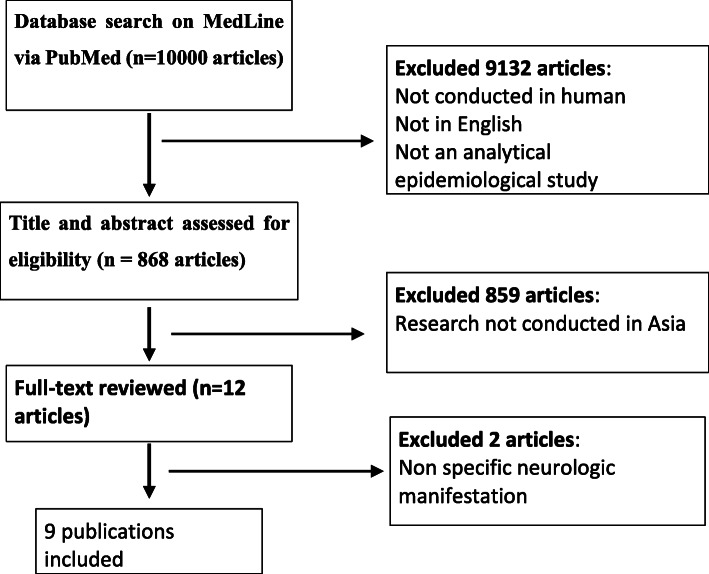
Flow diagram of the stages of study selection for systematic review

The PRISMA statement is used in the process of writing this review [3]. We performed a systematic review based on searched database from MEDLINE online databases (accessed via PubMed) that were published from December 2019 to September 2020 with the latest 1 year publication, according to the time of first COVID-19 case to the latest month of WHO global situation report published. This article used PICO model for eligibility criteria and applied to each research found in the database. We searched published articles in the online database systematically by applying the following keywords, (Neurologic manifestation) OR (Neurological manifestation) OR (Central nervous system) OR (Peripheral nervous system) OR (Focal neurologic deficit) OR (Global neurological deficit) AND (COVID-19)) OR (SARS Cov-2) OR (Coronavirus) OR (Coronavirus-2) OR (Novel Coronavirus) OR (Coronavirus Disease2019) AND (Confirmed case) OR (Suspected case). All the keywords were searched with all study design, excluding the systematic review and meta-analysis and restricted only in English. We then screened for the title of the publication that was performed in Asian region with neurologic manifestation of COVID-19 infection. The diagram flow of this review can be seen in Fig. 1 [3].

## Results

After applying the search method, we found a total of 9 retrieved articles from the electronic database to answer our research question of the neurologic manifestation in COVID-19 infection in Asia. Among these 9 articles, 5 of them are case report and 1 case series, 1 prospective multi-center cohort study, 1 retrospective multi-center study, and 1 retrospective observational study. All articles reported confirmed COVID-19, confirmation by positive swab test using the real time reversed transcriptase polymerase chain reaction (RT-PCR) method, with neurologic manifestation, disorder or syndrome on presentation or found during hospital stay. In case of neurologic disorder or syndrome, we categorized them into encephalitis and ADEM, acute cerebrovascular disease, acute symptomatic seizure and Guillain-Barré syndrome. All extracted data were summarized in Table 1. Also, Table 2 will describe the comparison of number of neurological disorders to number of total patients included.

**Table 1:** Neruologic manifestation of COVID-19 infection according to publications included

Author	Time and place of the study	Study methods	Patient/population	Neurologic disorder/symptoms	Neurologic manifestations	Supporting examination results
Koh JS, and colleagues [4]	2020/Singapore	Prospective multi-center cohort study	47,572 confirmed COVID-19 patients (84.4% mild symptoms or asymptomatic, 2.2% severe symptoms and 13.3% critical); median age 34 years old; 98% male	4 patients with ADEM/encephalitis; 25 patients with Acute ischemic stroke or TIA or intracerebral hemorrhage; 7 patients with mono and polyneuropathies 4 patients with dysautonomia	Loss of consciousness, spastic paralysis (*quadriparesis*), transient ocular flutter, right and left *hemiplegia*/*hemiparesis*	**CSF** (various results), **brain MRI** (white matter lesion, brainstem lesion, spinal cord lesion), multi-focal hemorrhagic lesion predominantly in white matter
Mao and colleagues [5]	2020/China	Retrospective, observational study	214 confirmed COVID-19 patients; average age 52.7 years old; 87 (40.7%) men	Acute ischemic stroke (4 patients), intracerebral hemorrhage (1 patient)	**CNS symptoms**: altered consciousness, headache, dizziness, sudden onset *hemiplegia*, convulsive seizure and ataxia **PNS symptoms**: taste, smell and vision impairment, neuropathic pain	—
Lu and colleagues [6]	2020/China	Retrospective multi-center study	304 people (discharged or died from COVID-19)	Acute symptomatic seizure	Generalized tonic-clonic seizure	**EEG** (not routine due to exposure concern)
Chandrasekaran and colleagues [7]	2020/Philippines	Case report	62 years old, female (history of hypertension, prediabetes, dyslipidemia and TIA)	Acute ischemic stroke	Sudden dysarthria; sudden right side *hemiparesis*	**Brain CT scan** (hypodensity of left corona radiata); **CT-angiography** (left M1-segment of MCA stenosis)
Kamal YM and colleagues [8]	2020/United Arab Emirates	Case report	31 years old, man, previously healthy	Encephalitis	Physical and verbal aggressivity; altered mental status; abnormal behavior	**Non-contrast brain CT scan** (multiple hypodensities in the external capsule bilaterally, insular cortex and deep periventricular white matter of frontal lobe bilaterally); **brain MRI with contrast** (hyperintensity lesion of temporal lobe bilaterally with involvement of parasagittal frontal lobes bilaterally in FLAIR and T2-weighted image)
Khalifa and colleagues [9]	2020/Palestine	Case report	11 years old boy	Guillain-Barré syndrome	Acute unsteady gait, inability to walk and climb stairs; Symmetrical weakness of lower limb (strength 3/5), hypotonia, ankle and knee *areflexia*; tingling sensation in legs and feet bilaterally, impaired proprioception	**Whole spine MRI with contrast** (enhancement of *cauda equina* nerve roots); **nerve conduction study** (consistent with demyelination polyneuropathy)
Wang and colleagues [10]	2020/China	Case report	68 years old man	Mental abnormalities associated with COVID-19 with CNS and PNS symptoms	Loss of consciousness, headache, dizziness; trembling of the hands, neck stiffness, muscle weakness (4/5 strength); persecution delusion	**Brain CT scan** (lacunar lesion in left basal ganglia)
Kwon and colleagues [11]	2020/South Korea	Case series	59 years old male (hypertension, diabetes, CKD); 84 years old male (hypertension, hyperlipidemia, pulmonary tuberculosis); 65 years old male (chronic heart disease); 87 years old female (hypertension, AF, heart failure); 83 years old male (hypertension, CKD)	Acute ischemic stroke	Sudden onset *Hemiparesis*, dysarthria, altered mental status	Brain CT scan, Brain MRI, CT-angiography, MR-angiography (2 patients right MCA lesion; 2 patients left MCA lesion; 1 unknown result)
Wada and colleagues [12]	2020/Japan	Case report	69 years old man (diabetes mellitus)	Guillain-Barré syndrome	Loss of consciousness; Ankle and knee *hyporeflexia*, *paraparesis inferior* (strength 4/5)

**Table 2:** Comparison of neurological manifestation/disorder to total patients of each study

Author	Study methods	Neurological manifestation/disorder	Number of patients with neurological manifestation/disorder	Total number of patients
Koh JS and colleagues [4]	Prospective multi-center cohort study	Acute disseminated encephalomyelitis (ADEM) and encephalitis	4 (4.4%)	90 patients
		Cerebrovascular disorder (AIS/TIA)	19 (21.1%)	
		Cerebral venous thrombosis	4 (4.4%)	
		Intracerebral hemorrhage	2 (2.2%)	
		Peripheral nervous system disorder (mono/polyneuropathy)	7 (7.8%)	
		Dysautonomia	4 (4.4%)	
Mao and colleagues [5]	Retrospective, observational study	Dizziness	36 (16.8%)	214 patients
		Headache	28 (13.1%)	
		Impaired consciousness	16 (7.5%)	
		Acute cerebrovascular disease	6 (2.8%)	
		Ataxia	1 (0.5%)	
		Seizure	1 (0.5%)	
		Taste impairment (dysgeusia/ageusia)	12 (5.6%)	
		Smell impairment (hyposmia/anosmia)	11 (15.1%)	
		Vision impairment	3 (1.4%)	
		Nerve pain	5 (2.3%)	
Lu and colleagues [6]	Retrospective multi-center study	Acute symptomatic seizure	304 (100%)	304 patients
Chandrasekaran and colleagues [7]	Case report	Acute ischemic stroke	1 (100%)	1 patient
Kamal YM and colleagues [8]	Case report	Encephalitis	1 (100%)	1 patient
Khalifa and colleagues [9]	Case report	Guillain-Barré syndrome	1 (100%)	1 patient
Wang and colleagues [10]	Case report	Mental abnormalities associated COVID-19	1 (100%)	1 patient
Kwon and colleagues [11]	Case series	Acute ischemic stroke	5 (100%)	5 patients
Wada S and colleagues [12]	Case report	Guillain-Barré syndrome	1 (100%)	1 patient

### Neurologic manifestation findings on encephalitis in COVID-19 infection

In this study, we found two reports of encephalitis. One report from Koh JS and colleagues in Singapore with 4 severe encephalitis cases in confirmed case of COVID-19 [4]. These patients have median interval of 24 days into first onset of encephalitis symptoms since first showing COVID-19-related symptoms. The first case showed spastic quadriparesis and transient ocular flutter [4]. The other one, case number 3, had two episodes of hemiplegia on the right and left side of the body, while the other 2 had no focal neurologic deficit [4]. The CSF analysis examination showed mild pleocytosis, and raised protein, while case number 2 showed normal result. Case number 3 and 4’s CSF were not examined [4]. Brain MRI also conducted in this report, which showed findings consistent with ADEM in case 1, multi-focal hemorrhagic lesions predominantly in the white matter in case 4 and encephalitis in case 2 and case 3 [4]. Cases 1, 2, and 4 eventually recovered after corticosteroid and corticosteroid-IVIG combination therapy [4]. Case 3 died 3 months into illness [4].

Another report from Kamal and colleagues, on the case of encephalitis in COVID-19 confirmed patient. A case report of a 31-year-old, previously healthy man without fever, presented to the emergency with impairment of mental state and behavioral abnormality [8]. On the neurologic examination, the patient is severely agitated and level of consciousness was fluctuated [8]. Brain CT without contrast were conducted and showed multiple hypodensity lesion in the bilateral external capsule, deep periventricular white matter of the frontal lobe bilaterally, and the insular cortex [8]. Two weeks after, brain MRI with contrast were conducted and showed hyperintensity lesion in the bilateral temporal lobe symmetrically with bilateral parasagittal frontal lobes involvement, suggestive of encephalitis [8]. The patient was treated with symptomatic and empirical treatment for COVID-19 infection based on the guideline of the country and also 750 mg of acyclovir for the encephalitis for 2 weeks [8]. One week after admission, despite the fluctuating confusion and agitation, the patient’s level of consciousness dramatically improved. Eventually, the patient regains full conscious and coherence with complete resolution of his agitation and able to resume normal life routine [8].

### Neurologic manifestation findings on acute cerebrovascular disease in COVID-19 infection

Four studies reported acute cerebrovascular disease, which are transient ischemic attack (TIA), acute ischemic stroke, intracerebral hemorrhage (ICH), and cerebral venous thrombosis (CVT). On the study by Koh and colleagues, there were 25 patients with acute cerebrovascular disease with 16 acute ischemic stroke (AIS), 3 patients had TIA, 2 patients had intracerebral hemorrhage, and 4 patients had CVT [4]. All of these 25 patients were tested positive for COVID-19 using RT-PCR method. Twelve of 19 AIS and TIA patients were asymptomatic for COVID-19 and 3 others had critical COVID-19 [4]. The stroke subtype of 14 radiologically confirmed (brain MRI) acute ischemic stroke out of 16 patients are small-vessel stroke in 5 patients, 2 patients cardioembolic, 2 patients large-vessel, 2 patients cryptogenic stroke, and 3 other undetermined etiology [4]. An IV thrombolysis therapy was administered to 1 patient and the other 2 patients was treated with mechanical thrombectomy. Two patients with intracerebral hemorrhage were all on critical condition of COVID-19 with multi-organ failure [4]. Both suffered massive ICH and died. Four cases of CVT all occurred in young men of age 27–39 years old. Two of them were asymptomatic while the other two had mild symptoms of COVID-19 [4]. All 4 patients had unilateral thrombosis sigmoid and transverse sinuses, extended into jugular vein in 3 patients, 1 of whom suffered a temporal lobe hematoma and another 1 had near complete thrombosis of superior sagittal sinus [4]. Three of them received anticoagulation therapy and 2 of them recovered, 1 died despite anticoagulation therapy [4]. The other one was not given anticoagulation therapy due to subdural hematoma [4].

A study by Mao and colleagues, reported 5 cases of acute cerebrovascular disease, with 4 case of acute ischemic stroke and 1 intracerebral hemorrhage. All of these occurred in the patients with severe COVID-19 infection group in the study [5]. These symptoms developed early in the illness with 1–2 days of median time [5]. Two cases of 5 acute cerebrovascular disease case presented with sudden hemiplegia but asymptomatic to COVID-19-related symptoms [5].

Another case of acute cerebrovascular disease was reported by Chandrasekaran and colleagues, in a case report of a 62-year-old female, with hypertension, prediabetes, dyslipidemia, and history of TIA 1 year prior to the onset [7]. This patient presented with sudden onset of right-side hemiparesis and dysarthria and with the score of National Institutes of Health Stroke Scale (NIHSS) 4 [7]. A brain CT scan resulted in a hypodensity lesion of the left corona radiata and centrum semiovale, and the CT-angiography showed significant stenosis of the M1 branch of the middle cerebral artery (MCA) [7].

A case series by Kwon and colleagues, reported five cases of acute cerebrovascular disease. The first case is a 59-year-old man, hypertensive, diabetic, with chronic kidney disease (CKD), and previous ischemic stroke history, presented with a sudden right hemiparesis and dysarthria. The brain CT scan showed normal findings. This patient is also confirmed SARS-CoV-2 infection by RT-PCR. On the 3rd day, the symptoms developed into an acute left side hemiparesis [11]. The second case is an 84-year-old man with hypertension and hyperlipidemia, presented 10.7 h after the onset with sudden altered mental status. The brain MR-angiography confirmed an occlusion of the M1 branch of right MCA and acute infarction in the right MCA territory. He also confirmed for SARS-CoV-2 infection, with RT-PCR, but was asymptomatic [11]. Case number 3 is a 65-year-old man, 7 days onset of COVID-19-related symptoms (cough, fever, and chest pain). He was treated with mechanical ventilation in the intensive care unit (ICU). On the 13th day, he developed a new onset of sudden right-side weakness. The craniocerebral CT showed multiple hypodensity lesion in both left posterior cerebral artery (PCA) and MCA territories. The transthoracic echocardiogram (TTE), revealed a 5 mm in size vegetation on the mitral valve. The TTE was conducted after this patient was suspected for cardioembolic etiology after a normal CT-angiography [11]. The fourth case is an 87-year-old woman with history of heart failure, atrial fibrillation, and hypertension, presented with 3 h onset of aphasia and right hemiparesis. The brain MR-angiography and MRI showed acute multiple infarction in the last MCA territory [11]. The last case is a case of 83-year-old man, with CKD, hypertension, and currently on routine aspirin, presented 24.7 h with sudden dysarthria and right arm weakness. The brain MRI confirmed acute multiple infarctions in the left MCA territory [11].

### Neurologic manifestation findings on Guillain-Barré syndrome in COVID-19 patients

We found two study reported Guillain-Barré syndrome (GBS) with one case in each report. The first case was reported by Khalifa and colleagues in a case report of a 11-year-old boy in Palestine complained of acute onset of inability to walk or climb stairs accompanied by unsteady gait and associated with tingling sensation in both lower limb (feet and legs) [9]. This patient had an acute URTI with low-grade fever 3 weeks before the onset of unsteady gait presented [9]. On neurologic examination, Khalifa and colleagues found symmetrical weakness of lower limb with 3/5 muscle strength, lost ankle and knee reflexes, hypotonia, tingling sensations and impaired sensation of light touch and pain of both feet up to mid-legs [9]. MRI with contrast of the brain and whole spine was conducted and showed enhancement of the *cauda equina* nerve roots. The nerve conduction study also showed result of demyelinating polyneuropathy matching the clinical presentation of GBS [9]. The patient then treated with intravenous immunoglobulin at 1 g/kgBW/day for 2 days and improved on gait, balance, lower limb muscle power and decreased numbness, and normal proprioception [9].

The second case was reported by Wada and colleagues, a case report of a 69-year-old man with diabetes mellitus, presented with 17 days onset of diminished deep tendon reflexes and decreased consciousness [12]. There is also slight muscle weakness of both lower limbs. Ten days later, the patient presented with fever and was diagnosed with SARS CoV-2 infection causing pneumonia, after chest CT and swab test with RT-PCR method showed positive result for SARS CoV-2 infection [12]. CSF analysis showed cytoalbuminologic dissociation. There was no other supporting examination conducted and the patient was treated with Intravenous immunoglobulin at 0.5 g/kgBW/day for 6 days [12]. The patient’s symptoms improved and was discharged after two negative result confirmed, with ability to walk independently and speak normally [12].

### Neurologic manifestation findings on acute symptomatic seizure in COVID-19 infection

A study by Lu and colleagues reported acute symptomatic seizure in the patients with COVID-19 infection in China. They reported that the most common risk factors for seizure is hypoxia and mostly happened in patient with severe case of COVID-19 (*n* = 77) with 63 severe case compared to 14 people with mild case [6]. The were 304 people included in this study with 59.9% male and 108 severe cases out of 304 [6]. There were no long term nor electroencephalogram (EEG) recorded due to exposure concerns.

### Other neurologic manifestation on COVID-19 infection

In the study reported by Koh and colleagues, other than mentioned above, they also reported other neurologic manifestation, such as unilateral facial neuropathy in 5 patients and 4 cases of dysautonomia [4]. The four patients with dysautonomia has a median interval of 8 days between onset of COVID-19 [4]. The dysautonomia symptoms are acute persistent symptomatic orthostatic tachycardia confirmed with autonomic function test (AFT), which showed marked tachycardia on standing and passive 60^0^ tilt without hypotension and hyperhidrosis [4]. The other three patient developed isolated acute near vision difficulty with asymmetrical accommodation defects, within 2 weeks of illness onset. Their dysautonomia also confirmed with AFT [4].

Mao and colleagues also reported other neurologic manifestation other than mentioned above which is divided into central nervous system (CNS) group and peripheral nervous system (PNS) group. In CNS group, the most common are dizziness in 36 patients (16.8%) and headache in 28 patients (13.1%) [5]. The other CNS manifestations are ataxia in one patient (0.5%) and seizure in one patient (0.5%) [5]. The PNS manifestations are taste impairment in 12 patients (5.6%) and smell impairment in 11 patients (5.1%) and nerve pain in 5 patient (2.3%) [5]. According to this study, neurologic manifestation were significantly more common in severe case of COVID-19 infection compared to mild or asymptomatic COVID-19 manifestation. They also reported that those with CNS manifestation, lymphocytes levels, platelet counts, and higher blood urea nitrogen (BUN) levels are lower compared to those without CNS manifestation [5]. Whereas for patients with PNS manifestation, there were no significant differences in laboratory findings compared to patients without PNS manifestation [5].

A case report by Kamal and colleagues and Wang and colleagues both reported mental abnormalities accompanying neurologic manifestation related to COVID-19 infection. In the case report by Kamal and colleagues, the patient was diagnosed with encephalitis, but presented with physical and verbal aggressiveness and later developed altered mental status and behavioral changes [8]. The first craniocerebral CT, without contrast, revealed multiple hypodense lesion in the insular cortex and the deep periventricular white matter of the frontal lobes bilaterally and external capsules bilaterally.

The case report by Wang and colleagues reported a 68-year-old man presented with loss of consciousness and low fever accompanied by headache and dizziness [10]. A brain CT revealed lacunar lesion in the left basal ganglia region [10]. The patient woke up 48 h later and since then was treated for 2 weeks for his COVID-19 [10]. During his treatment, he once again slipped into comatose state and 4 days later woke up with inability to walk, with uroclepsia, coprolalia, and persecution delusion [10]. Repeated brain CT showed similar result to the previous one [10]. A CSF analysis was done and revealed significantly high protein level (803.6 mg/l), but no obvious abnormalities was found in routine examination [10]. He was then discharged with slight shaking of both hands, mild irritability, and walking fatigue [10].

## Discussion

Our review results show various neurologic manifestation in patients with confirmed COVID-19 infection which shows that there is an involvement in the pathophysiology of COVID-19 infection to the brain structure. However, as SARS-CoV-2 infection is still a new disease, the mechanism of the brain and its structures involvement is still widely studied. From the results, it is showed that the most reported neurologic manifestation associated with COVID-19 is acute cerebrovascular disease. The most well-known mechanism of the cerebrovascular disease in COVID-19 might be due to a coagulopathy. SARS-CoV-2 infection can cause damage to endothelial cells, it will then activate thrombotic and inflammatory pathway [13]. Activated thrombotic pathways and monocyte will contribute to cause secondary hemophagocytic lymphohistocytosis described in the pathophysiology of severe COVID-19 [14]. The dysfunction of endothelial caused by coagulopathy potentially lead to macrovascular and microvascular complications of the brain [15]. Early inflammatory process can also cause acute ischemic stroke as it destabilize a carotid plaque e or trigger atrial fibrillation [13].

There is also some case of encephalitis and acute disseminated encephalomyelitis (ADEM). SARS-CoV-2, as with other neurotropic viruses, can enter the brain through the olfactory bulb, which is the only part of the CNS not protected by dura, which then contribute to the anosmia symptoms in COVID-19. This entry route is thought to be the route for herpes simplex virus which is the most common organism causing a sporadic viral encephalitis [15]. Alternative entry route include carriage through infected leukocytes or across the blood-brain barrier (BBB) [13]. The angiotensin-converting enzyme-2 (ACE-2) receptor that bind with SARS-CoV-2 to enter the cell is found in smooth muscle and vascular endothelial of the brain [13]. More specifically, the SARS-CoV-2 utilizes the ACE-2 as its entry receptor and not only ACE-2, but also TMPRSS2 cell protease is utilized by the virus to prime its S protein [16]. The ACE-2/TMPRSS2 co-expression in oligodendrocytes, which found by surveying the human tissue ACE-2 and TMPRSS2 positive cell, could be the causes of CNS infiltration or proliferation [16]. There is a study of transgenic mice infected with SARS-CoV-2 that expressed human ACE-2 receptors showed susceptibility to the virus, more efficient replication of the virus as compared to wild-type mice, more detectable viral antigen in the brain, more severe pulmonary lesions, cerebral vasculitis and hemorrhages [17]. It can be understood that by downregulating the expression of ACE-2, SARS-CoV-2 will disrupt the delicate balance of ACE/ACE-2 cerebrovascular control which resulted in unopposed ACE signal, disrupted cerebral auto-regulation or excessive vasoconstriction. There is other mechanism of infection related to SARS-CoV infection which is associated with high levels of cytokines, including interleukin (IL)-1β, IL-6, IL-12, interferon gamma (INFγ) and tumor necrosis factor-alpha (TNFα) [18]. There can be disruption of the integrity of the BBB by immune-mediated toxicity and cytokine-driven injury in the absence of direct viral spread [16]. Cytokines may also be mediate or even inhibit injury to cells of the CNS alone or acting in synergy, due to directly neurotoxic [19]. But, the inflammatory pathways that explain the ways in which the highly activated cytokine signaling in SARS-CoV-2 impact neurologic outcome are not fully understood yet.

There is also reports of Guillain-Barré syndrome (GBS) related to COVID-19 infection. Many infectious agents, including cytomegalovirus, human immunodeficiency virus (HIV), ZiKa virus, Epstein-Barr virus, and *Campylobacter jejuni* have been associated with GBS. In this review, we found 2 report of GBS case related to COVID-19, one is a case of 11-year-old boy and another one is a case of 69-year-old man. The reported incidence of GBS ranges from 0.4 to 1.4 cases per 100,000 children per year and from 1 to 2 cases per 100,000 adults with most patients are elderly men [20]. SARS-CoV-2 virus are thought to cause GBS in certain patients either directly through ACE-2 receptors on neuronal tissue and neuroinvasive capacity, or indirectly through the response of the immune system [21]. A study indicated that SARS-CoV-2 infection is able to cause an immune reaction with an increased level of interleukin-6 (IL-6) which stimulates the inflammatory cascade and damage tissues. Therefore, inflammatory factors played a more important role in this case [21].

The limitation of this review is that among 9 databases included, 6 of them are case report and case series. As case report and case series are not often used in systematic review because it cannot generalize findings. The inclusion of case reports and case series in this review is because COVID-19 is still a new disease and the research of the disease is still developing. Therefore, further review including more article other than case report and/or case series is needed.

## Conclusion

COVID-19 is an infection caused by SARS-CoV-2 virus infection. Its main infected organ is the respiratory system, which is the main entry route of the infection. COVID-19 can cause various infection symptoms, including fever, dry cough, shortness of breath, chest pain, fatigue, and many more. It is well known that COVID-19 also affect other organ, including the brain, which may result in a global or focal neurologic manifestation. Healthcare provider treating patient with COVID-19 infection should also be aware of neurologic manifestation associated with COVID-19 infection so an early suspicion and diagnosis of COVID-19 can be made and improve patient outcome.

## Data Availability

No applicable.

## Abbreviations

COVID-19: Coronavirus disease
RT-PCR: Reversed transcriptase polymerase chain reaction
SARS: Severe acute respiratory syndrome
WHO: World Health Organization
PICO: People, Intervention, Control, Outcome
PRISMA: Preferred Reporting Items for Systematic Reviews and Meta-Analyses
ADEM: Acute disseminated encephalomyelitis
TIA: Transient ischemic stroke
CSF: Cerebrospinal fluid
MRI: Magnetic resonance imaging
CNS: Central nervous system
PNS: Peripheral nervous system
EEG: Electroencephalogram
CT: Computerized tomography
CKD: Chronic kidney disease
AF: Atrial fibrillation
MCA: Middle cerebral artery
IVIG: Intravenous immunoglobulin
ICH: Intracerebral hemorrhage
CVT: Cerebral venous thrombosis
NIHSS: National Institutes of Health Stroke Scale
AIS: Acute ischemic stroke
TTE: Transthoracic echocardiogram
ICU: Intensive care unit
GBS: Guillain-Barré syndrome
URTI: Upper respiratory tract infection
AFT: Autonomic function test
BUN: Blood urea nitrogen
ACE: Angiotensin-converting enzyme
TMPRSS2: Transmembrane protease serine 2
BBB: Blood-brain barrier
IL: Interleukin
INF: Interferon
TNF: Tumor necrosis factor
HIV: Human immunodeficiency virus
